# Marital and Romantic Satisfaction Among Older African Americans

**DOI:** 10.1093/geroni/igab046.184

**Published:** 2021-12-17

**Authors:** Antonius Skipper, Robert Taylor

**Affiliations:** 1 Georgia State University, Atlanta, Georgia, United States; 2 University of Michigan, Ann Arbor, Michigan, United States

## Abstract

There remains a lack of knowledge on marital satisfaction of African Americans generally, but particularly older African Americans. In addition, only a handful of studies investigate satisfaction among couples who are unmarried. With data from the National Survey of American Life, this study examined the correlates of romantic and marital satisfaction among older African Americans. Findings reveal that married older African Americans were slightly more satisfied with their relationship than individuals who were either remarried or unmarried but in a romantic relationship. Among older African American married adults, older age was associated with higher marital satisfaction, and men had higher levels of marital satisfaction than women. Also, married older African Americans with lower family incomes reported higher marital satisfaction. Given the limited research on older African Americans couples, either married or unmarried, this study offers valuable implications for individuals and professionals engaging these couples in practical settings.

